# The PCAT3/PCAT9-miR-203-SNAI2 axis functions as a key mediator for prostate tumor growth and progression

**DOI:** 10.18632/oncotarget.24198

**Published:** 2018-01-12

**Authors:** Fangfang Tao, Xinxin Tian, Zhiqian Zhang

**Affiliations:** ^1^ Department of Immunology and Microbiology, Basic Medical College, Zhejiang Chinese Medical University, Hangzhou 310053, Zhejiang, People's Republic of China; ^2^ Department of Biochemistry and Biophysics, Texas A and M University and Texas AgriLife Research, College Station, TX 77843-2128, USA; ^3^ Tianjin International Joint Academy of Biomedicine (TJAB), Tianjin 300457, People's Republic of China; ^4^ State Key Laboratory of Medicinal Chemical Biology, Nankai University, Tianjin 300071, People's Republic of China

**Keywords:** prostate cancer, LncRNAs, PCAT3, PCAT9, miR-203-SNAI2 axis

## Abstract

Long non-coding RNAs (lncRNAs) have been reported to be of great importance in the formation and progression of a wide range of human carcinomas including prostate cancer (PCa). Among them, PCAT3 and PCAT9 have been identified as two prostate tissue-specific lncRNAs and are up-regulated in PCa. However, their roles in the biological characteristics of PCa have not been fully elucidated. In the present study, our data revealed that knockdown of PCAT3 and PCAT9 suppressed cellular proliferation, invasion, migration, angiogenesis and stemness in androgen-dependent LNCaP and 22Rv1 cells. Strikingly, bioinformatics analysis predicted that both PCAT3 and PCAT9 transcripts had two conserved binding sties for miR-203. Meanwhile, dual luciferase report assays revealed that miR-203 could suppress the luciferase activities of reporter plasmids carrying the binding site of miR-203 on the mRNA of PCAT3 or PCAT9. Quantitative RT-PCR (qRT-PCR) and RNA fluorescence *in situ* hybridization (RNA-FISH) showed that miR-203 mimic reduced the expression of PCAT3 and PCAT9 both in LNCaP and 22Rv1 cells. We also noted that both PCAT3 and PCAT9 inhibited miR-203 expression and alleviated repression on the expression of SNAI2, a critical regulator of epithelial-mesenchymal transition directly targeted by miR-203. Functionally, silence of miR-203 or ectopic expression of SNAI2 attenuated the inhibitory effect of PCAT3 and PCAT9 knockdown on cell proliferation and migration *in vitro*, and xenograft growth *in vivo*. Taken together, our data suggested that the PCAT3/PCAT9-miR-203-SNAI2 axis may serve as a promising diagnostic and therapeutic target for PCa.

## INTRODUCTION

Prostate cancer (PCa) is one of the most common epithelial carcinomas and the second leading cause of cancer-related death after lung cancer among men worldwide [1, 2]. Prostate carcinogenesis and progression are multi-step processes involving multiple genetic mutations and gene deregulation [3, 4]. Accumulating evidence has demonstrated that deregulation of specific genes, including microRNAs (miRNAs) and long non-coding RNAs (lncRNAs), is responsible for the pathogenesis and progression of PCa [5, 6]. Nonetheless, the cross-talk between miRNAs and lncRNAs in PCa remains poorly understood. Defining the mechanisms of miRNA-lncRNA network occurred in PCa will not only advance our knowledge of the nature of PCa, but also will help identify the therapeutic targets for the treatment of PCa.

Long non-coding RNAs (lncRNAs), which belong to a class of non-protein coding transcripts longer than 200 nucleotides in length, have been implicated in various biological processes, including tumor development and metastasis and regulate protein expression at transcriptional, post-transcriptional and translational levels. A wide range of literatures have reported that aberrant expression of lncRNAs can be indicative of certain stages of PCa and plays an important role in the pathogenesis and progression of PCa. PCAT3, also known as PCA3, is one of the most specific prostate cancer diagnostic biomarker involved in controlling prostate cancer cell surviving and modulating androgen receptor signaling [7–10]. PCAT9, also known as PCGEM1, is also one of highly prostate-specific lncRNAs in PCa regulated by androgen [11–13]. However, the function of PCA3 and PCAT9 in prostate cancer progression, including migration, angiogenesis, and cancer-related stemness, has not been fully understood.

MiRNAs are a non-coding class of short single strand RNAs of approximately 20–24 nucleotides in length and post-transcriptionally repress target gene expression by binding to the 3′ untranslated regions (3′-UTRs) of protein-coding mRNAs. There is no doubt that miRNAs play essential roles in the regulation of a variety of physiological and pathological processes, including tumorigenesis. Growing functional evidence supports the critical role of miRNAs in the development of PCa. For example, tumor suppressor miRNAs, including let-7 family, miR-200 family, miR-203, miR-205, miR-15a/miR-16-1, miR-101, miR-449, miR-99 family, miR-126*, miR-145, miR-146a, miR-224, miR-330 and miR-34a, and oncogenic miRNAs, including miR-221, miR-222, miR-21, miR-32, miR-141, miR-18a and miR-125b, have been identified as prostate cancer-associated miRNAs [14–24]. miR-203 has been reported to be downregulated in prostate cancer and associated with cancer metastasis by targeting BMI1, LASP1, ZEB2 and SNAI2 [25–29]. Although broadly characterized as a tumor suppressor in PCa, to our knowledge, data on the upstream regulatory mechanism of miR-203 in PCa have not yet been well elucidated.

As a member of the SNAI/SLUG superfamily, SNAI2 has been implicated in the pathogenesis of different cancers. In PCa, SNAI2 promotes prostate cancer xenograft growth via modulating Cyclin D1 [30]. Meanwhile, SNAI2 knockdown in PCa suppresses the activity of cancer stem cells by reducing the expression of multiple pluripotent genes [31–33]. Several studies have also showed that the miR-203/SNAI2 axis plays a broad-spectrum role in various types of cancers [34–38]. However, the cross-talk among lncRNAs, miRNAs and SNAI2 in the formation and progression of prostate cancer has not been reported.

Importantly, lncRNAs have also been demonstrated to function as molecular sponges for miRNAs. For instance, Zhu M. et al. reported that lncRNA H19-miR-675 axis acts as a suppressor of prostate cancer metastasis by targeting TGFβ1 [39]. PCAT9 stimulates proliferation of osteoarthritic synoviocytes by acting as a sponge for miR-770 [40]. In the present study, we demonstrate that PCAT3 and PCAT9 regulates tumorigenesis, migration, angiogenesis, stemness and metastasis in prostate cancer via modulating the miR-203/SNAI2 axis. These findings provide a potential novel diagnostic and therapeutic target for PCa.

## RESULTS

### Silence of PCAT3 and PCAT9 suppresses cell growth, invasion and migration in LNCaP and 22Rv1 cells

**Figure 1:**
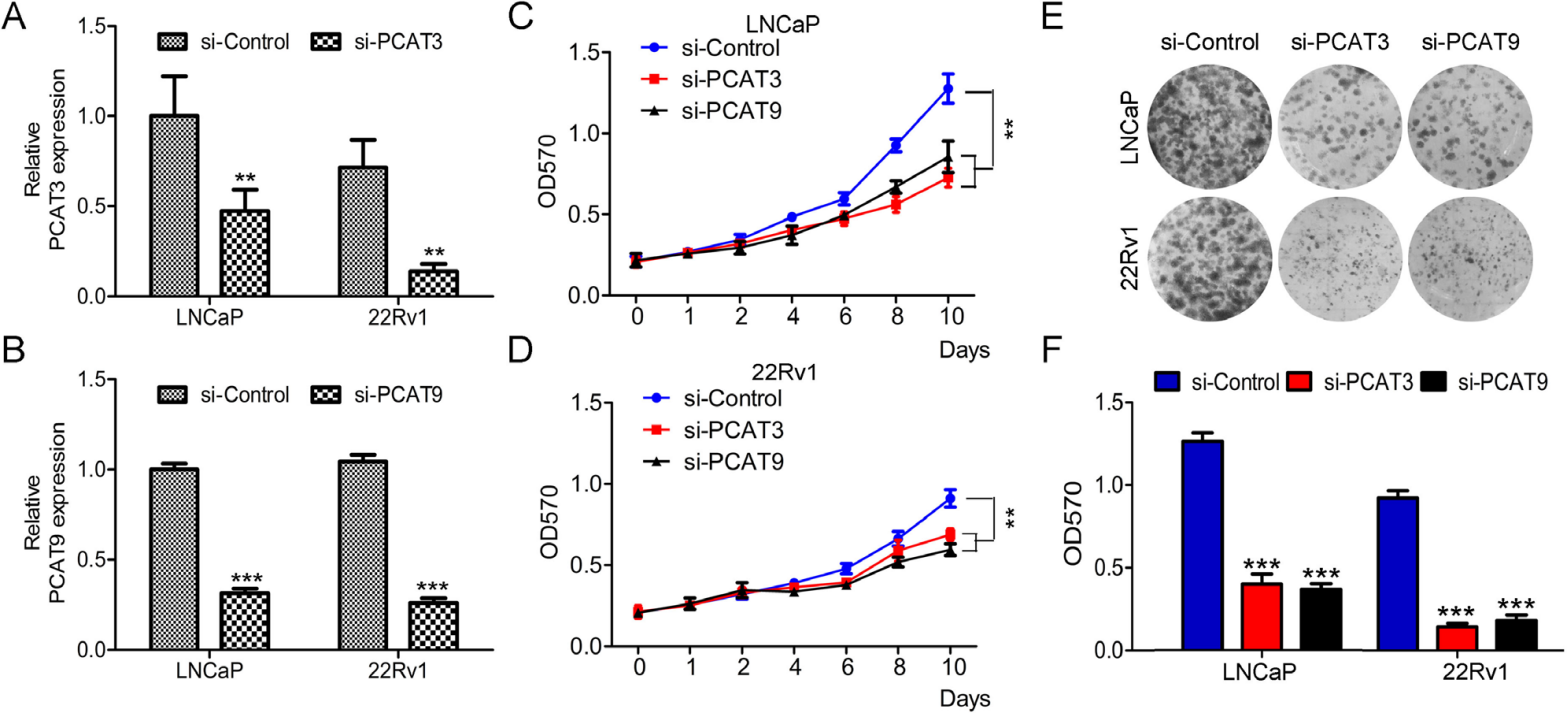
LncRNAs PCAT3 or PCAT9 knockdown suppresses prostate cancer LNCaP and 22Rv1 cell proliferation (**A** and **B**) Quantitative RT-PCR to detect the expression of PCAT3 and PCAT9 in LNCaP (A) and 22Rv1 (B) cells transfected with PCAT3 or PCAT9 siRNA. (**C** and **D**) The relative cell viability and growth rate of LNCaP (C) and 22Rv1 (D) cells transfected with PCAT3 or PCAT9 siRNA, which was determined by MTT assay. Data were shown as means ± SEM, *n* = 6. (**E** and **F**) Silence of PCAT3 and PCAT9 in LNCaP and 22Rv1 cells significantly decreased the soft agar growth and colony formation. Quantitation was performed by measurement of absorbance at OD570 for resolved crystal violet. Data are means ± SEM. *n* = 3.

**Figure 2:**
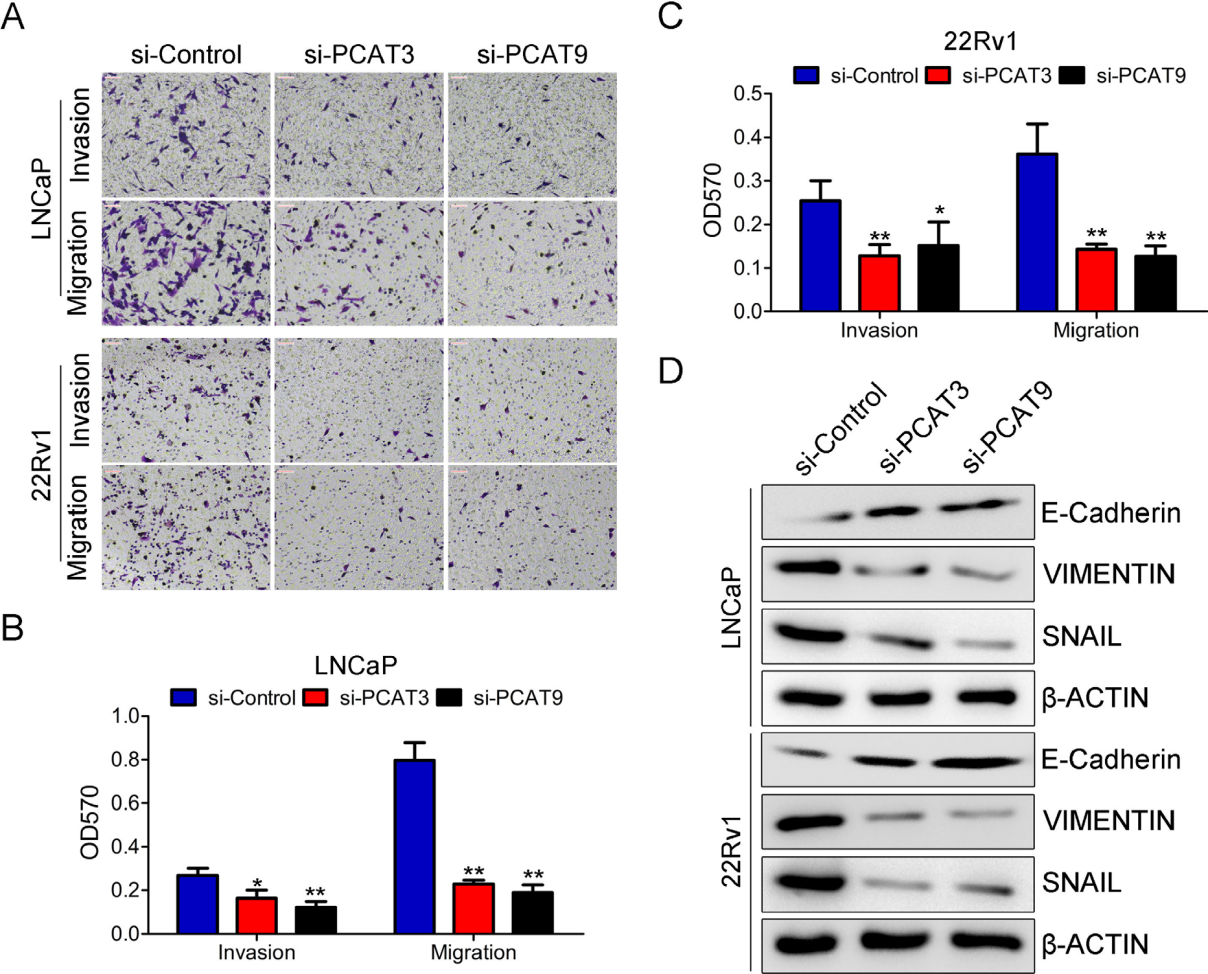
Silence of PCAT3 or PCAT9 significantly inhibits migration of LNCaP and 22Rv1 cells (**A**) Representative images to present cell migration ability for LNCaP and 22Rv1 cells transfected with control siRNA, PCAT3 siRNA or PCAT9 siRNA. Scale bar: 100 µm. (**B** and **C**) The cell migration ability was qualified by measurement of absorbance at OD570 for resolved crystal violet. Data are means ± SEM. *n* = 3. (**D**) Western blot analysis to determine the expression of E-CADHERIN, VIMENTIN and SNAIL in LNCaP and 22Rv1 cells transfected with PCAT3 of PCAT9 siRNA.

The primary goal of this study was to determine the potential role of androgen-dependent lncRNAs PCAT3 and PCAT9 in PCa. To this end, we used LNCaP and 22Rv1 cells which are androgen-sensitive. Cells were transfected with PCAT3 or PCAT9 siRNA for 48 h prior to soft agar colony formation assay, MTT assay and transwell migration assay. Transfection efficiency was determined by quantitative RT-PCR (Figure 1A and 1B). As depicted in Figure 1C and 1D, loss of function of PCAT3 or PCAT9 led to significant decrease in LNCaP and 22Rv1 cell proliferation compared to the negative control. Similar observation was seen in the colony formation assay whereby the silence of PCAT3 or PCAT9 suppressed the clonogenic potential both in LNCaP and 22Rv1 cells (Figure 1E and 1F). In addition, down-regulation of PCAT3 or PCAT9 showed a significant reduction in cellular invasion and migration (Figure 2A–2C). Meanwhile, silence of PCAT3 or PCAT9 also obviously suppressed the expression of mesenchymal markers VIMENTIN and SNAIL and elevated the expression of epithelial maker E-CADHERIN both in LNCaP and 22Rv1 cells (Figure 2D). These findings indicate that PCAT3 and PCAT9 play a pro-oncogenic role in prostate carcinogenesis and progression.

### Silence of PCAT3 or PCAT9 suppressed tumor-driven angiogenesis and cancer-associated stemness in PCa

**Figure 3:**
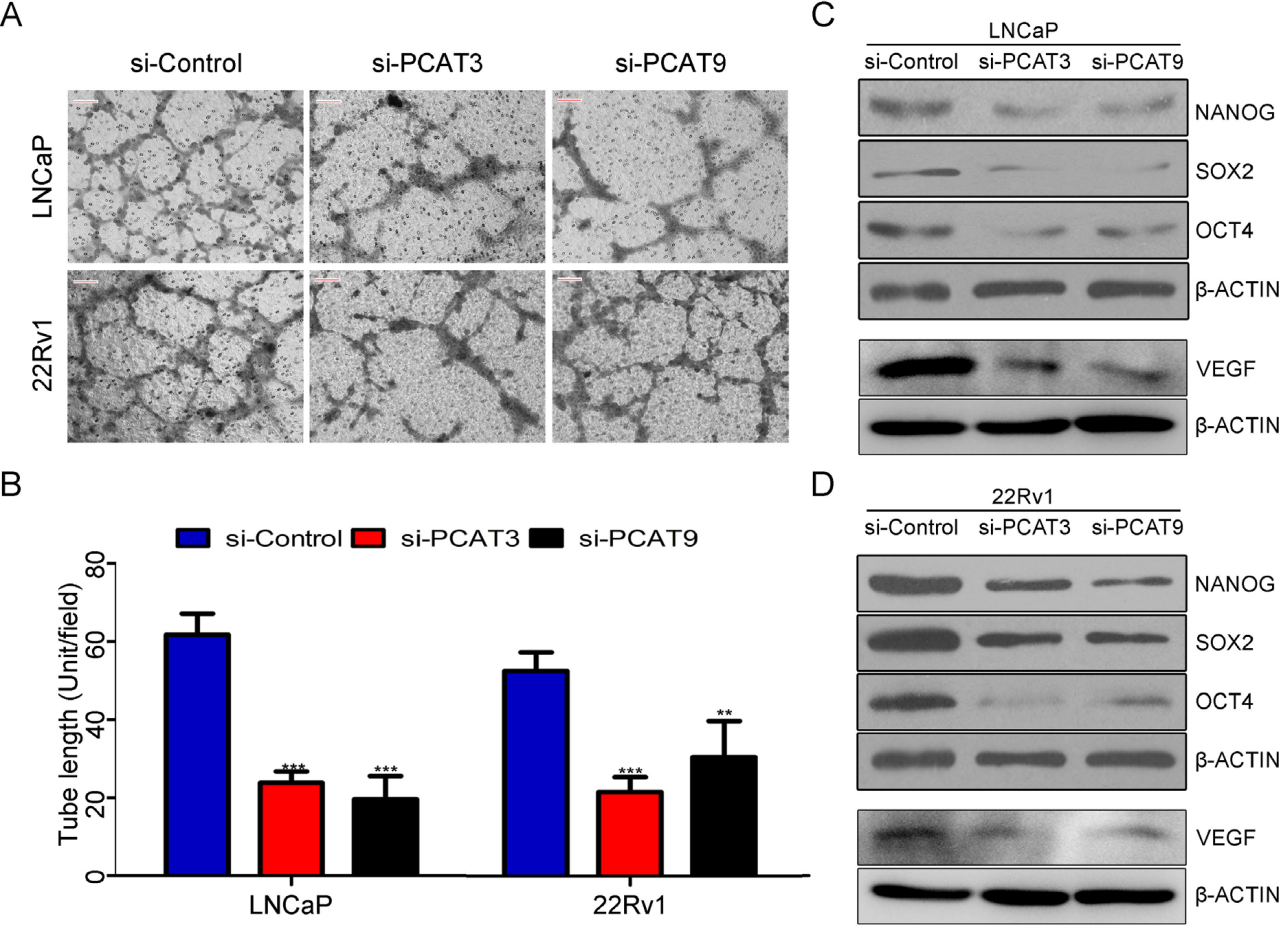
Loss of function of PCAT3 or PCAT9 in LNCaP and 22Rv1 cells reduced endothelial cell vasculature formation and suppresses stemness associated factors (**A** and **B**) Vasculature formation assay (A) of HuVECs with conditioned media from LNCaP and 22Rv1 cells transfected with control siRNA, PCAT3 siRNA or PCAT9 siRNA. For vasculature formation assay, tube length (B) was measured with ImageJ software. Scale bar: 100 µm. (**C** and **D**) Western blot to determine the expression of NANOG, SOX2, OCT4 and VEGF in LNCaP (C) and 22Rv1 (D) cells transfected with control siRNA, PCAT3 siRNA or PCAT9 siRNA.

Tumor-driven angiogenesis and cancer stemness have been reported to be of great importance in tumor progression. Next, we examined whether knocking-down endogenous PCAT3 or PCAT9 expression in PCa could modulate vasculature formation of endothelial cells. LNCaP and 22Rv1 cells were transfected with control siRNA, PCAT3 siRNA or PCAT9 siRNA for 48 hours. Conditioned cell culture media were collected and added to HuVECs for vasculature formation assays. As shown in Figure 3A and 3B, vasculature formation were obviously reduced when stimulated with conditioned media from LNCaP and 22Rv1 cells transfected with PCAT3 or PCAT9 siRNAs, compared with control siRNA. In addition, SOX2, OCT4 and NANOG are transcription factors that play key roles in maintaining the pluripotency of embryonic stem cells and cancer stem cells. Western blot assay showed that silence of PCAT3 or PCAT9 decreased the protein levels of OCT4, SOX2, NANOG and VEGF in LNCaP and 22Rv1 cells, suggesting that PCAT3 and PCAT9 may modulate the properties of angiogenesis and cancer stem cells in PCa (Figure 3C and 3D). In addition, we also detected the expression of VEGF in the tumors with PCAT3 of PCAT9 siRNA transfection and found that silencing of PCAT3 or PCAT9 could significantly suppress the expression of VEGF (Supplementary Figure 1). These data further confirmed the critical role of PCAT3 and PCAT9 in aggressive PCa.

### Reciprocal repression of PCAT3 and PCAT9 with miR-203 in PCa

**Figure 4:**
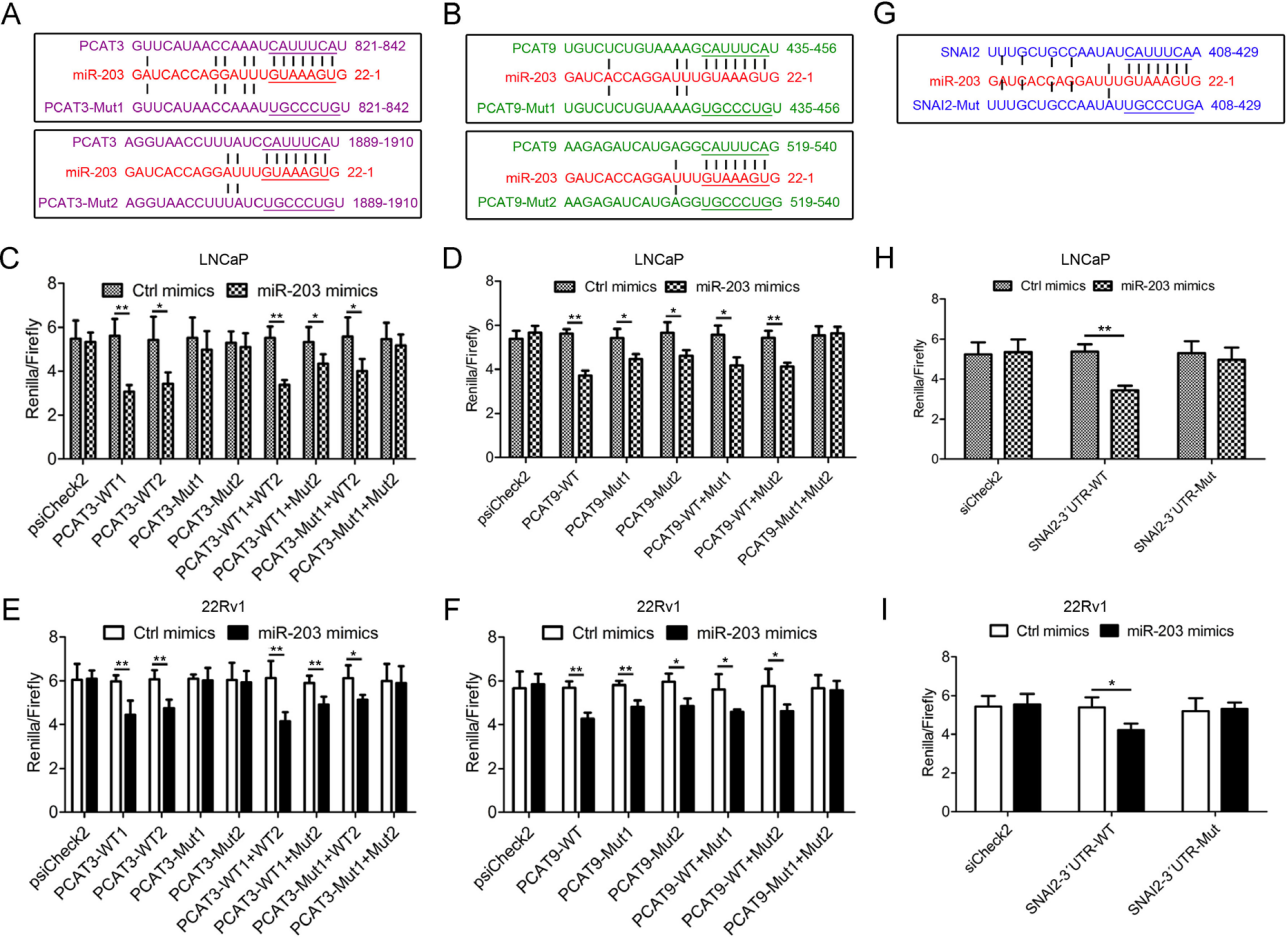
Reciprocal repression of PCAT3 and PCAT9 with miR-203 in PCa (**A**, **B** and **G**) Putative miR-203-binding sites in the mRNAs of PCAT3 (A) and PCAT9 (B), and the 3′-UTR of SNAI2 (G). The reporter constructs showed the wild type (WT) binding sites and the mutated sequences. (**C**, **D**, **E** and **F**) miR-203 mimic transfection suppressed the luciferase activities of reporter plasmids carrying the binding site of miR-203 on the mRNA of PCAT3 or PCAT9. (**H** and **I**) Luciferase reporter assays were utilized to examine the interaction between miR-203 and SNAI2 3′-UTR using reporters containing the 3′-UTR region of SNAI2.

To address the regulatory mechanism of PCAT3 and PCAT9 in prostate carcinogenesis and progression, we used the online software starBase v2.0 (http://starbase.sysu.edu.cn/) to identify the miRNAs potentially interacting with PCAT3 and PCAT9. StarBase v2.0 predicted that both PCAT3 and PCAT9 contain two conserved binding sequences complementary to miR-203 seed regions (Figure 4A and 4B). Meanwhile, the luciferase report assay demonstrated that miR-203 mimic reduced the luciferase activities of reporter plasmids carrying the binding site of miR-203 on the mRNA of PCAT3 or PCAT9, but the change was diminished in the mutant reporter (Figure 4C–4F), suggesting that miR-203 can function as a sponge to repress PCAT3 and PCAT9 in PCa. In our previous studies and some other literatures, the transcription factor SNAI2 have been reported to be a direct target of miR-203 in multiple cancers (Figure 4G). Herein, we also performed a luciferase report assay to determine the effect of miR-203 on SNAI2 expression in LNCaP and 22Rv1 cells. As expected, the luciferase report assay indicated that miR-203 mimic inhibited the luciferase activity of the wild type SNAI2 3′-UTR reporter (SNAI2 3′-UTR-WT), but no change was observed for the luciferase activity in the mutant reporter (SNAI2 3′-UTR-Mut) (Figure 4H and 4I).

**Figure 5:**
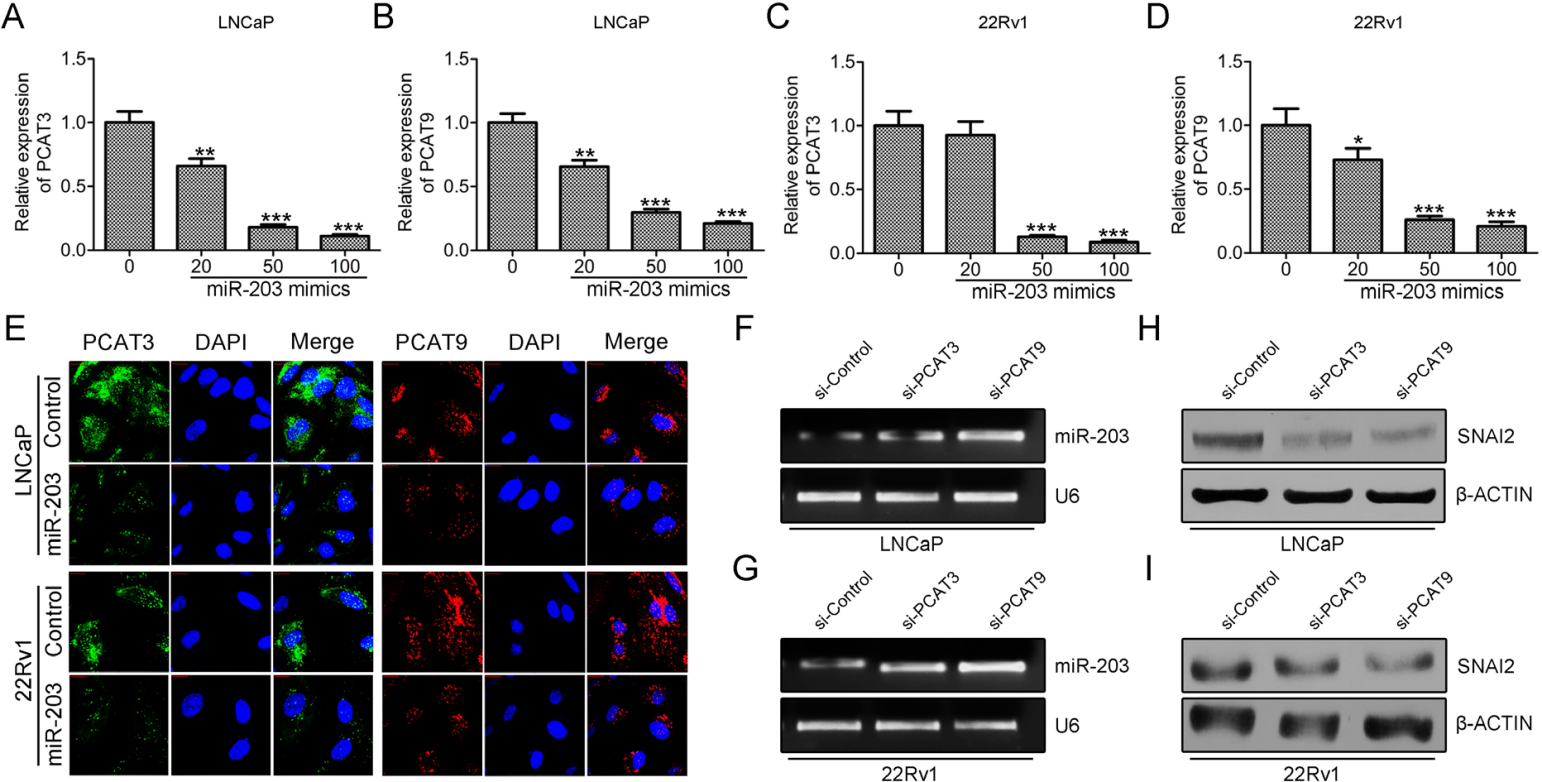
Both PCAT3 and PCAT9 function as decoys for the miR-203-SNAI2 axis (**A**, **B**, **C**, **D** and **E**) Detection of PCAT3 and PCAT9 expression using qRT-PCR (A–D) and RNA-FISH (E) in LNCaP and 22Rv1 cells transfected with miR-203 mimic. Scale bar: 20 µm. (**F** and **G)**. Semi quantitative-RT-PCR to detect the expression of miR-203 in LNCaP (F) and 22Rv1 (G) cells transfected with control siRNA, PCAT3 siRNA or PCAT9 siRNA. (**H** and **I)**. Western blot to detect the expression of SNAI2 in LNCaP (H) and 22Rv1 (I) cells transfected with control siRNA, PCAT3 siRNA or PCAT9 siRNA.

Moreover, the expression of PCAT3 and PCAT9 was significantly decreased in cells transfected with miR-203 mimic, which was confirmed by qRT-PCR (Figure 5A–5D) and RNA-FISH (Figure 5E). Additionally, we found that the expression level of miR-203 was up-regulated in LNCaP and 22Rv1 cells transfected with PCAT3 or PCAT9 siRNA, compared with control siRNA (Figure 5F and 5G). Meanwhile, the protein level of SNAI2 was suppressed by PCAT3 siRNA and PCAT9 siRNA (Figure 5H and 5I). Therefore, we conclude that both PCAT3 and PCAT9 form a reciprocal repression regulatory loop with miR-203 to regulate prostate cancer proliferation and progression by modulating SNAI2 expression.

### PCAT3 and PCAT9 promote tumor cell proliferation and progression in PCa by sponging the miR-203-SNAI2 pathway

**Figure 6:**
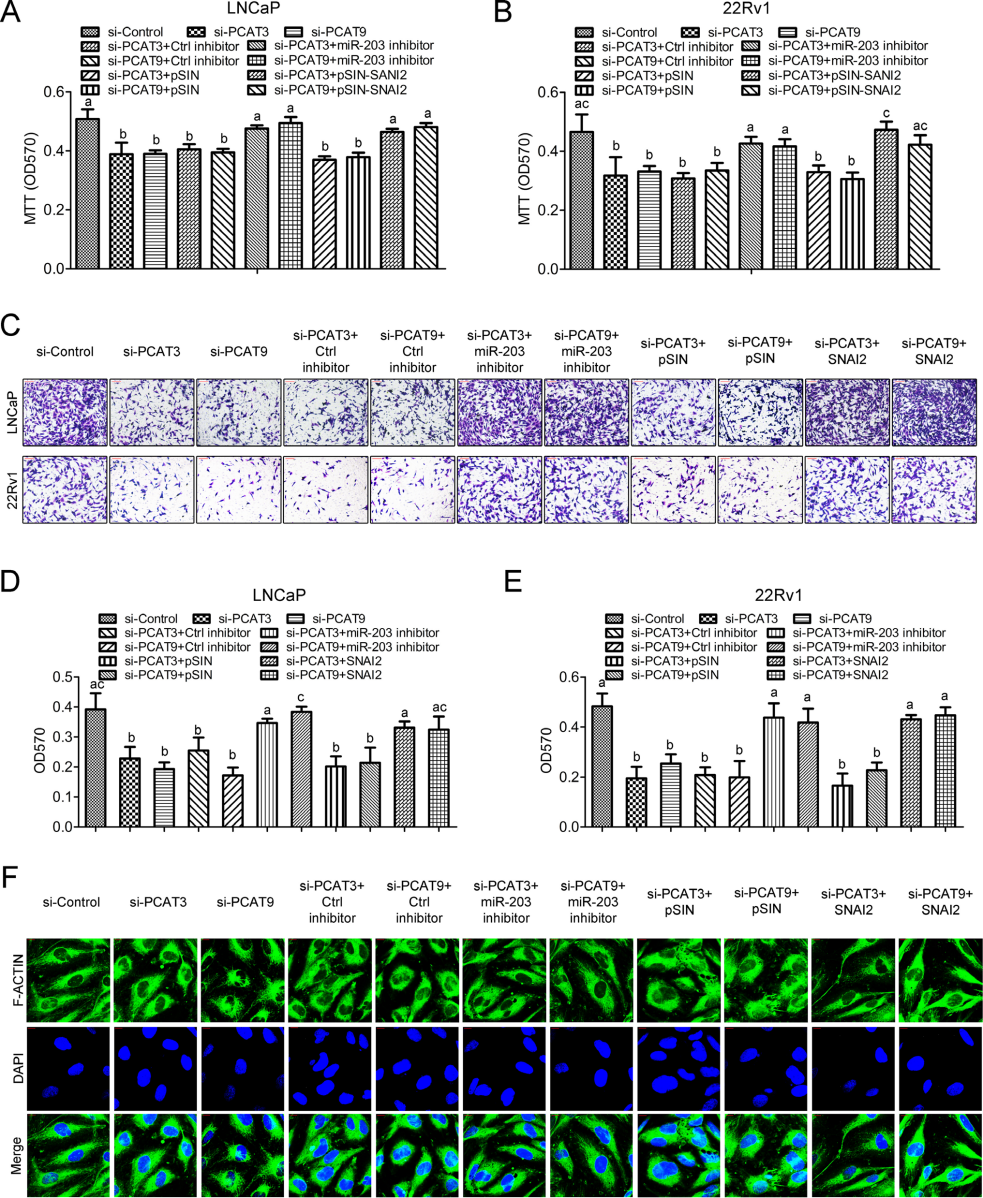
The PCAT3/PCAT9-miR-203/SNAI2 regulatory axis regulates prostate cancer cell proliferation and migration (**A**) MTT assay was performed to determine the cell viability of LNCaP (A) and 22Rv1 (**B**) in the indicated groups. (**C**, **D** and **E**) The cell migration ability in the indicated groups was determined (C) and qualified (D and E) using transwell migration assay. Scale bar: 100 µm. (**F**) Detection of F-actin using immunofluorescence in LNCaP cells with indicated treatment. Scale bar: 20 µm. Different letters above the bars indicate statistically significant difference with *p* < 0.05.

To further determine the role of PCAT3/PCAT9-miR-203-SNAI2 in prostate cancer proliferation and progression, rescue experiments were conducted. The groups were shown in Figure 6: control siRNA, PCAT3 siRNA, PCAT9 siRNA, PCAT3 siRNA plus control miRNA inhibitor, PCAT9 siRNA plus control miRNA inhibitor, PCAT3 siRNA plus miR-203 inhibitor, PCAT9 siRNA plus miR-203 inhibitor, PCAT3 siRNA plus pSIN empty lentivirus, PCAT9 siRNA plus pSIN empty lentivirus, PCAT3 siRNA plus pSIN-SNAI2 lentivirus or PCAT9 siRNA plus pSIN-SNAI2 lentivirus were transfected or infected into LNCaP and 22Rv1 cells to investigate their effects on cell proliferation. MTT assay indicated that inhibition of miR-203 or overexpression of SNAI2 significantly alleviated the suppressive effect of PCAT3 siRNA or PCAT9 siRNA on cell viability (Figure 6A and 6B). Moreover, transwell migration assay demonstrated that silence of miR-203 or SNAI2 overexpression rescued the migration ability of LNCaP and 22Rv1 cells suppressed by PCAT3 or PCAT9 siRNA (Figure 6C–6E). Similar observation was seen in the F-actin staining whereby the LNCaP cell shape in the groups control siRNA, PCAT3 siRNA + miR-203 inhibitor, PCAT9 siRNA + miR-203 inhibitor, siRNA + pSIN-SNAI2 lentivirus and PCAT3 siRNA + pSIN-SNAI2 lentivirus was more elongated than cells in other groups (Figure 6F).

### The PCAT3/PCAT9-miR-203-SNAI2 axis participates in the regulation of xenograft growth in PCa *in vivo*

**Figure 7:**
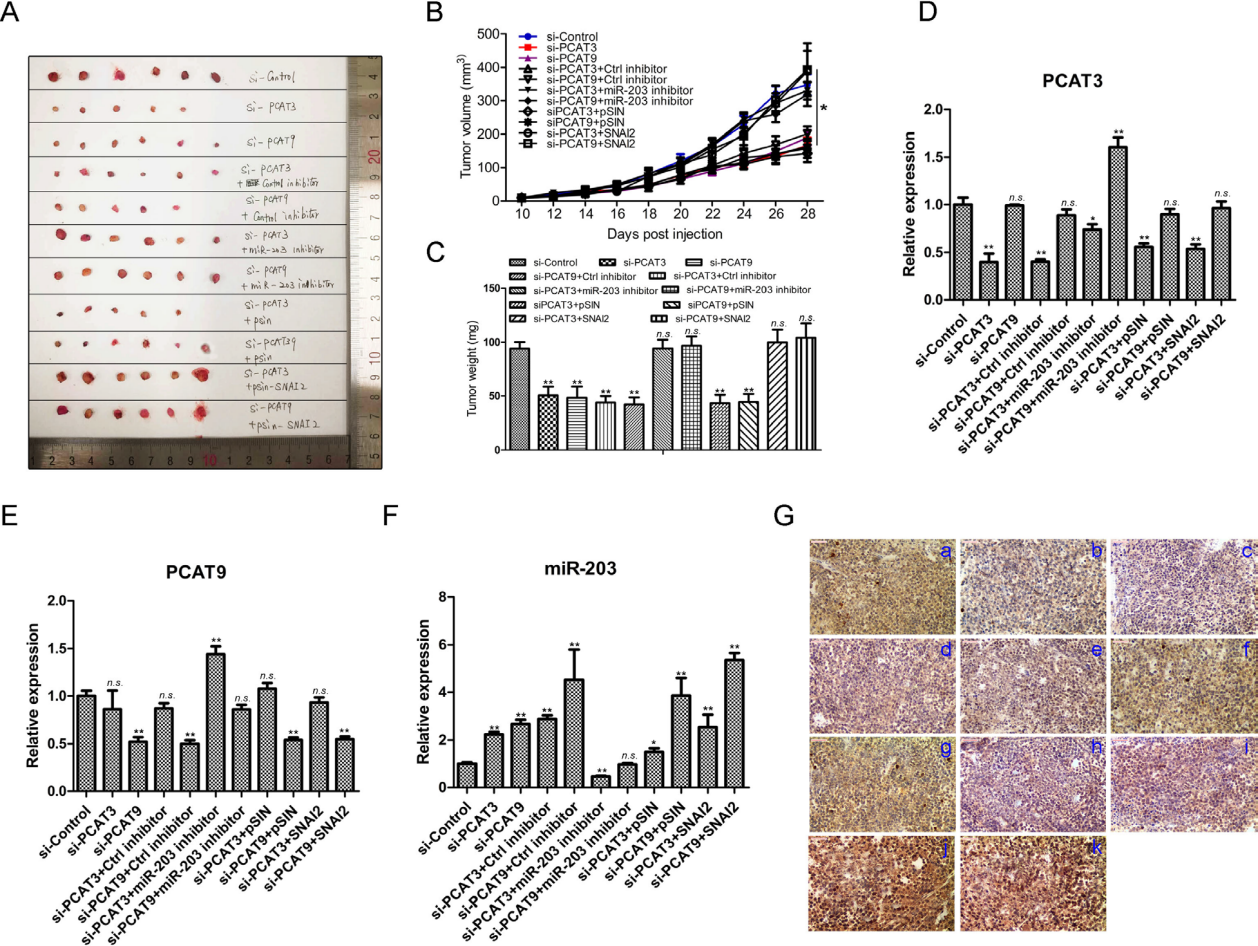
The PCAT3/PCAT9-miR-203/SNAI2 axis controls xenograft growth of LNCaP cells *in vivo* (**A**) Representative pictures of tumors from LNCaP xenografts in the groups of control siRNA, PCAT3 siRNA, PCAT9 siRNA, PCAT3 siRNA + control inhibitor, PCAT9 siRNA + control inhibitor, PCAT3 siRNA + miR-203 inhibitor, PCAT9 siRNA + miR-203 inhibitor, PCAT3 siRNA + pSIN, PCAT9 siRNA +pSIN, PCAT3 siRNA + pSIN-SNAI2 or PCAT9 siRNA + pSIN-SNAI2, respectively. (**B** and **C**) Tumor growth curve (B) and Tumor weight (C) of LNCaP xenografts in the indicated groups. Tumor volumes and weight represented as means ± SD. *n* = 6. (**D**–**F**) Quantitative RT-PCR to determine the average expression levels of PCAT3, PCAT9 and miR-203 in the tumors in indicated groups. (**G**) Representative images to show the expression of SNAI2 in the xenografts in indicated groups, which was determined by immunohistochemistry. Scale bar: 100 µm. a: control siRNA; b: PCAT3 siRNA; c: PCAT9 siRNA; d: PCAT3 siRNA plus control inhibitor; e: PCAT9 siRNA plus control inhibitor; f: PCAT3 siRNA plus miR-203 inhibitor; g: PCAT9 siRNA plus miR-203 inhibitor; h: PCAT3 siRNA plus pSIN; i: PCAT9 siRNA plus pSIN; j: PCAT3 siRNA plus pSIN-SNAI2; k: PCAT9 siRNA plus pSIN-SNAI2.

To further delineate the *in vivo* function of PCAT3/PCAT9-miR-203-SNAI2 axis in PCa, xenograft tumor model were established in BALB/C nude mice. LNCaP cells were transfected with control siRNA, PCAT3 siRNA, PCAT9 siRNA, PCAT3 siRNA plus control inhibitor, PCAT9 siRNA plus control inhibitor, PCAT3 siRNA plus miR-203 inhibitor, PCAT9 siRNA plus miR-203 inhibitor, PCAT3 siRNA plus pSIN, PCAT9 siRNA plus pSIN, PCAT3 siRNA plus pSIN-SNAI2 or PCAT9 siRNA plus pSIN-SNAI2 were injected subcutaneously into the upper area of the hind limb. Once the tumors were palpable, measurements were performed each other day. As expected, xenografts transfected with PCAT3 or PCAT9 siRNA grew at a much slower rate than the control siRNA group (Figure 7A). Obvious reductions in tumor size and weight were observed at the termination of experiment (*p* < 0.01) (Figure 7B and 7C). In addition, the xenograft growth rate was significantly rescued by miR-203 inhibitor or pSIN-SNAI2 lentivirus. These results suggest the miR-203-SNAI2 axis may be of great importance in PCAT3/PCAT9 guided prostate tumor growth *in vivo*. In addition, the transfection efficacy and regulations among PCAT3, PCAT9, miR-203 and SNAI2 in the xenograft tissues were confirmed by quantitative RT-PCR or immunohistochemistry (Figure 7D–7G).

**Figure 8:**
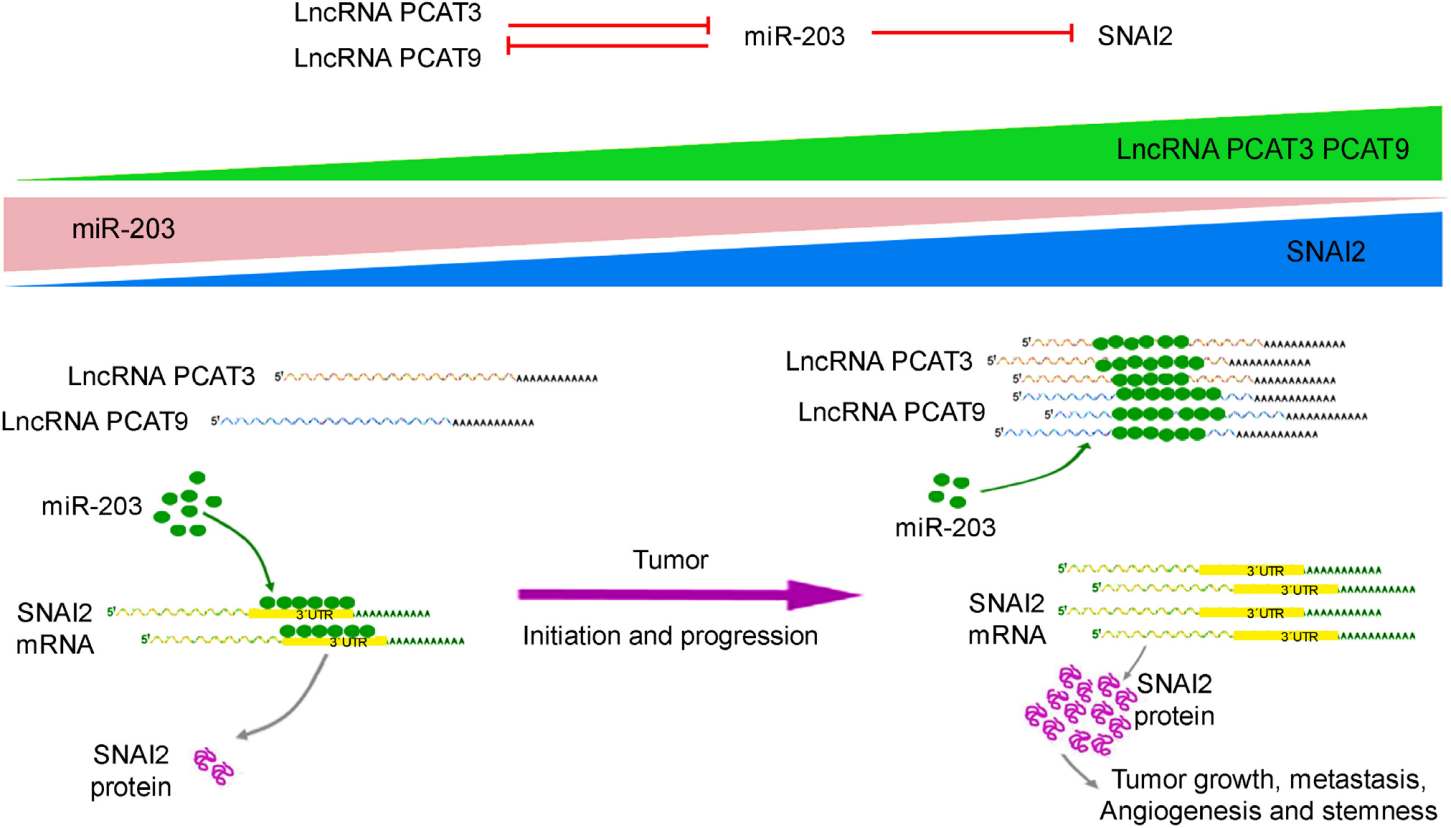
Schematic representation to show the expression and function of the PCAT3/PCAT9-miR-203-SNAI2 axis in prostate cancer Upon tumor development, a reciprocal suppression between miR-203 and lncRNAs PCAT3/PCAT9 regulates the transcription and translation of SNAI2. p53 mainly acts as a transcriptional activator and to a minor extent as a transcriptional repressor. The differential expression of PCAT3/PCAT9, miR-203 and SNAI2 leads to the induction of specific cellular processes, such as cell hyperproliferation, or epithelial-mesenchymal transition and angiogenesis, which promote tumor growth and progression.

In summary, our data systematically show that the PCAT3/PCAT9-miR-203-SNAI2 axis play a pivotal role in tumorigenesis, migration, angiogenesis and stemness in prostate cancer (Figure 8).

## DISCUSSION

Our previous studies and some other literatures have demonstrated that the miR-203-SNAI2 axis play a pivotal role in tumorigenesis [34–38]. However, it remains unclear whether and how lncRNAs act as regulators of this axis. In the present study, we demonstrated that both PCAT3 and PCAT9 could function as sponges to modulate the effect of miR-203-SNAI2 axis in PCa. Functionally, inhibition of miR-203 or overexpression of SNAI2 alleviated the suppressive effect of PCAT3 siRNA and PCAT9 siRNA on PCa proliferation, migration and xenograft growth. These data established a novel lncRNA-miRNA-transcription factor regulatory axis in PCa. As the lncRNAs PCAT3 and PCAT9 are prostate-specific non-coding transcripts, we naturally propose that this regulatory axis is also tissue-specific which only exists in PCa. Supporting this hypothesis, we also determined the expression of PCAT3 and PCAT9 in several breast cancer cell lines and tissues and no detectable signals in the qRT-PCR result (data not shown).

As a male hormone, androgen is essential for the development of the prostate as well as prostate cancer. Androgen deprivation therapy is regarded as a basic treatment for aggressive prostate cancer. PCAT3 and PCAT9 have been reported to be predominantly expressed in androgen-dependent prostate cancer cells and appear to be an essential component of androgen receptor (AR) signaling [7–11, 41, 42]. However, our unpublished data have demonstrated that the miR-203-SNAI2 axis play an important role in androgen-independent DU145 and PC3 cells. Herein, further studies will be needed to determine whether this axis is regulated by PCAT3 and PCAT9 in androgen-independent cells.

In addition, it is quite interesting to observe whether and how androgen modulates the PCAT3/PCAT9-miR-203-SNAI2 regulatory axis in PCa. Some literatures have shown that miR-203 and SNAI2 are related to androgen and involved in androgen receptor (AR) signaling pathways. For instance, Boll K et al. demonstrated that miR-130a, miR-203 and miR-205 jointly targeted several components of the mitogen-activated protein kinase (MAPK) and androgen receptor (AR) signaling pathways to interfere with progression to castration resistance [43]. Sun T et al. also identified seven miRNAs, including miR-221, miR-222, miR-23b, miR-27b, miR-15a, miR-16-1, and miR-203, which are differentially expressed in the androgen-sensitive LNCaP cells and the hormone resistant LNCaP-abl cells and hypothesized that these miRNAs may characterize certain subtypes of human castration resistant prostate cancer [44]. Wu K et al. have shown that SNAI2 is a unique androgen-regulated transcription factor and coordinates androgen receptor to facilitate castration resistance in prostate cancer [45]. In bladder cancer, SNAI2 mediated EMT has been reported to be critical for androgen-dependent metastasis [46]. In our future study, we will systematically elucidate the relationship between androgen and the PCAT3/PCAT9-miR-203-SNAI2 regulatory axis.

In our present study, it is the first time to demonstrate that the lncRNAs PCAT3 and PCAT9 play an important role in tumor-driven angiogenesis and cancer-related stemness. To support this conclusion, we examined the literatures about the relationship between androgen and tumor angiogenesis and stemness. It has been reported that angiogenesis is induced by elevated expression of vascular endothelial growth factor (VEGF) and VEGF is regulated by many factors in the tumor microenvironment including lowered oxygen levels and elevated androgens [47]. Thus, the regulation and function of VEGF in the PCAT3/PCAT9-miR-203-SNAI2 axis were also illustrated in this study. On the other hand, prostate cancer stem cells are characterized by heterogeneity, including AR+ and AR-, which are reflected in their response to hormone treatment [48]. Herein, it is much meaningful to determine the function of PCAT3/PCAT9-miR-203-SNAI2 axis in different types of prostate cancer stem cells in our future study.

In conclusion, this study allows us to demonstrate that lncRNA, miRNA and transcription factor can form a cascading network in tumorigenesis. Most importantly, deciphering the molecular mechanisms for the PCAT3/PCAT9-miR-203-SNAI2 axis linked to prostate cancer initiation and progression is of great importance for developing new diagnostic and therapeutic method.

## MATERIALS AND METHODS

### Cell cultures

Two prostate cancer LNCaP and 22Rv1 cells were purchased from the American Typical Culture Center (ATCC; Manassas, VA, USA) and cultured in RPMI 1640 (Gibco, Carlsbad, CA, USA) with 10% fetal bovine serum (FBS; Gibco) and 1% penicillin/streptomycin (Beyotime, Shanghai, China). Cells were incubated at 37°C in a humidified chamber supplemented with 5% CO_2_. Human umbilical vein endothelial cells (HuVECs) were purchased from Life Technologies (Carlsbad, California, USA) and cultured following the manufacturer’s protocols.

### siRNA transfection

SiRNAs against PCAT3 and PCAT9 were synthesized by Riobio Technology (Guangzhou, Guangdong, China). The siRNA sequences were as follows: 5′Phos/rCrUrArGrCrArCrArCrArGrCrArUrGrArUrCrArUrUrArCGG (si-PCAT3) and 5’Phos/rGrUrGrGrCrArArCrArGrGrCrArArGrCrArGrArGrGrGrAAA (si-PCAT9). Cells were transfected with siRNAs using lipofectamine RNAimax reagent (Invitrogen, Carlsbad, CA, USA) following the manufacturer’s protocol.

### Plasmid construction and lentiviral production

The open reading frame (ORF) of SNAI2 was obtained from the complementary DNA of DU145 cells and then cloned into the pSIN/EF1α-IRES-Puro lentiviral expression vector at the SpeI and EcoRI restriction sites. Lentivirus was produced by co-transfection of the pSIN expression vector, the packaging plasmid psPAX2 and the envelope plasmid pMD2.G into HEK-293T cells with Fugene HD (Roche, Basel, Switzerland). The primers used for cloning were as follows: 5′-GGACTAGTATGCCGCGCTCCTTCCTGGTC-3′ (pSIN-SNAI2, forward) and 5′-CGGAATTCTCAGTGTGCTACACAGCAGCCAGATTC-3′ (pSIN-SNAI2, reverse).

### MTT assay to determine cell viability

Treated cells were seeded in 96-well plates at a density of 2 × 10^3^ per well. After an incubation for indicated periods, 20 µl MTT solutions (Beyotime; 5 mg/ml) were added to each well for 4 h at 37°C. Then, the culture medium was removed and 200 µl of DMSO (Beyotime) were added to each well, and the plate was shaken gently for 10 min. Absorbance at 570 nm was measured using an ELISA reader (Moecular Devices, Sunnyvale CA, USA).

### Colony formation

Basic culture medium containing 0.6% agarose gel and 10% FBS was added to a 6-well plate and incubated at room temperature for 1 h. Forty hours after siRNA transfection, cells (5 × 10^3^) were re-suspended in RPMI 1640 medium containing 0.3% agarose gel and 10% FBS, and seeded into the pre-treated 6-well plate, followed by incubation for 14 days to allow colonies formation. The colonies were then washed twice with PBS, fixed with 70 % ethanol and stained with 0.1% crystal violet, and then the pictures were taken under a dissecting microscope a microscope (Olympus, Tokyo, Japan). Then, the stained crystal violet was resolved with 33% acetic acid and absorbance at 590 nm were determined. The data were performed in triplicate and showed as mean ± SD.

### Semi-quantitative RT-PCR and qRT-PCR

Semi-quantitative RT-PCR and qRT-PCR were performed as previously described [38]. The primers have been listed in the Table 1.

**Table 1:** Primers used in this study

Primer names	Sequences
PCAT3-forward	5**′**-AGATTTGTGTGGCTGCAGC-3′
PCAT3-reverse	5′-TCCTGCCCATCCTTTAAGG-3′
PCAT9-forward	5′-GGTAGGCACGTGGAGGACTA-3′
PCAT9-reverse	5′-TGCTTTTGTGGGTTTGTTCA-3
SNAI2-forward	5′-ATGAGGAATCTGGCTGCTGT-3′
SNAI2-reverse	5′-CAGGAGAAAATGCCTTTGGA-3′
specific RT primer for miR-203	5′-GTCGTATCCAGTGCAGGGTCCGAGGTATTCGCACTGGATACGACCTAGTG-3′
real time PCR, miR-203-forward	5′-GTGCAGGGTCCGAGGT-3′
real time PCR, miR-203-reverse	5′-GCCGCGTGAAATGTTTAGG-3′
real time PCR, U6-forward	5′-CTCGCTTCGGCAGCACA-3′
real time PCR, U6-reverse	5′-AACGCTTCACGAATTTGCGT-3
PCAT3-binding site-1-forward	5′-GACCGCGATCGCCCTTAAAGGATGGGCAGG-3′
PCAT3-binding site-1-reverse	5′-CTTAGTTTAAACCGTAATGATCATGCTGTGTG-3′
PCAT3-binding site-2-forward	5′-GACCGCGATCGCATCATCACATGAGACAGCAA-3′
PCAT3-binding site-2-reverse	5′-CTTAGTTTAAACGTGGTGATACATCATTGGCA-3′
PCAT9-binding site-forward	5′-GACCGCGATCGCTACTACGAGATGCACTGGGA-3′
PCAT9-binding site-reverse	5′-CTTAGTTTAAACAATCATATTTGCACACAACG-3′
SNAI2-3′-UTR-forward	5′-GACCGCGATCGCTGACAAATAAAGTCCAAAGGC-3′
SNAI2-3′-UTR-reverse	5′-CTTAGTTTAAACAATCATGAAGCAAGTAAAGTCTC-3′
PCAT3-binding site-1-mut-forward	5′-CGTTCATAACCAAATTGCCCTGTATTTCTAAC-3′
PCAT3-binding site-1-mut-reverse	5′-CAGGGCAATTTGGTTATGAACGCACAGTTTAG-3′
PCAT3-binding site-2-mut-forward	5′-AAGGTAACCTTTATCTGCCCTGTGGTGAGTGC-3′
PCAT3-binding site-2-mut-reverse	5′-CAGGGCAGATAAAGGTTACCTTTGGGGATTTG-3′
PCAT9-binding site-1-mut-forward	5′-CTGTCTCTGTAAAAGTGCCCTGTATTTACAAG-3′
PCAT9-binding site-1-mut-reverse	5′-CAGGGCACTTTTACAGAGACAGAGAATTTCA-3′
PCAT9-binding site-2-mut-forward	5′- AAAGAGATCATGAGGTGCCCTGGAGTGCACTG-3′
PCAT9-binding site-2-mut-reverse	5′- CAGGGCACCTCATGATCTCTTTTCCCTCTGC-3′
SNAI2-3′-UTR-mut-forward	5′-TTTTGCTGCCAATATTGCCCTGATCTGAAAAG-3′
SNAI2-3′-UTR-mut-reverse	5′-CAGGGCAATATTGGCAGCAAAAAAAAATGTA-3′

### Boyden chamber transwell migration and invasion assay

For invasion assay, 1 × 10^5^ cells were suspended into 200 µl of culture medium with 1% FBS and seeded into the upper chamber of a 24-well transwell insert (8 µm; BD Biosciences) pre-coated with 25 µl of growth-factor reduced matrigel (diluted into three volumes of serum-free culture medium). For migration assay, 5 × 10^4^ suspended cells were plated in the top chamber of a 24-well insert without pre-treatment. For both assays, fresh culture medium with 10% FBS was added into the lower chamber. After 24 h, the invaded or migrated cells were stained with 0.1% crystal violet and ten photographs were taken randomly for each sample. The stained crystal violet was resolved with 200 μl of 0.05 mM sodium citrate and 0.05 mM citric acid in 50% ethanol and measured at OD570 using an ELISA reader.

### Western blot analysis

Western blot analysis was performed as previously described [38]. The antibodies were as follows: anti-SNAI2 (Abcam, ab27568), anti-NANOG (Cell signaling technology, #3580), anti-SOX2 (Abcam, ab97959), anti-OCT4 (Cell signaling technology, #2750).

### Endothelial cell vasculature formation assay

Treated cells were seeded in 6-well dishes and cultured in 2 ml of medium containing 1% serum for another 48 hours to reach 100% confluence. Conditioned media were then collected, centrifuged at 1500 rpm/min, and the supernatant was used for endothelial cell vasculature formation assay. HuVECs (1 × 10^5^ per well) were seeded into a 24-well plate pre-coated with growth-factor reduced matrigel (BD Biosciences, San Jose, CA). After cultured for 8 hours with conditioned media, the tube structures were captured under the microscope (Olympus), and the extent of vasculature formation was qualified by measuring the cumulative tube length in a 10× field using ImageJ software. Five fields were examined to detect cell vasculature formation ability.

### Plasmid construction and Luciferase report assay

cDNA fragments of PCAT3 and PCAT9 and 3′-UTR fragment of SNAI2 with miR-203 binding sites were amplified from the cDNA and genomic DNA of LNCaP cells respectively and cloned into the downstream of the Renilla luciferase gene of the psiCHECK-2 vector (Promega, Fitchburg, WI, USA) at SgfI or PmeI sites. These constructs were then mutated using the Site-Directed Mutagenesis System (Beyotime) according to the manufacturer’s protocols. The primers used for cloning and site directed mutagenesis are listed in Table 1.

### Immunofluorescence assay

Immunofluorescence was performed as previously described [49]. *RNA-FISH* RNA-FISH was performed as previously described [50].

### *In vivo* experiment

All animal experiments were performed with the approval of the Animal Care and Use Center of TJAB. Male 4–6 weeks old BALB/c nude mice were used in this experiment. 1 × 10^7^ of LNCaP cells in the indicated groups (6 mice per group) were resuspended in PBS with 50% matrigel and subcutaneously injected to the back of the right lower limb. six mice were performed in each group. Tumor volumes were measured each other day after palpable tumors appeared. At 32 days post injection, mice were sacrificed, and tumors were surgically isolated, weighed and photographed.

### Statistical analysis

GraphPad Prism was applied for statistical analysis, which was determined with Student *t*-test. A value of *p* < 0.05 was considered significant. Asterisks in the figures represent as follow: ^*^p < 0.05, ^**^p < 0.01, and ^***^p < 0.001.

## SUPPLEMENTARY MATERIALS FIGURE



## Abbreviations

lncRNA: Long non-coding RNA
PCa: Prostate cancer
qRT-PCR: Quantitative RT-PCR
RNA-FISH: RNA fluorescence *in situ* hybridization
miRNA: MicroRNA
3′-UTRs: 3′ untranslated regions
FBS: Fetal bovine serum
HuVEC: Human umbilical vein endothelial cells
AR: Androgen receptor
MAPK: Mitogen-activated protein kinase
VEGF: Vascular endothelial growth factor

## References

[1] Nakao S, Mabuchi M, Shimizu T, Itoh Y, Takeuchi Y, Ueda M, Mizuno H, Shigi N, Ohshio I, Jinguji K, Ueda Y, Yamamoto M, Furukawa T (2014). Design and synthesis of prostate cancer antigen-1 (PCA-1/ALKBH3) inhibitors as anti-prostate cancer drugs. Bioorg Med Chem Lett.

[2] Husaini Y, Qiu MR, Lockwood GP, Luo XW, Shang P, Kuffner T, Tsai VW, Jiang L, Russell PJ, Brown DA, Breit SN (2012). Macrophage inhibitory cytokine-1 (MIC-1/GDF15) slows cancer development but increases metastases in TRAMP prostate cancer prone mice. PLoS One.

[3] Nagamatsu H, Teishima J, Goto K, Shikuma H, Kitano H, Shoji K, Inoue S, Matsubara A (2015). FGF19 promotes progression of prostate cancer. Prostate.

[4] Yokomizo A, Mai M, Bostwick DG, Tindall DJ, Qian J, Cheng L, Jenkins RB, Smith DI, Liu W (1999). Mutation and expression analysis of the p73 gene in prostate cancer. Prostate.

[5] Deng JH, Deng Q, Kuo CH, Delaney SW, Ying SY (2013). MiRNA targets of prostate cancer. Methods Mol Biol.

[6] He JH, Han ZP, Zou MX, Wang L, Lv YB, Zhou JB, Cao MR, Li YG (2017 Aug 24). Analyzing the LncRNA, miRNA, and mRNA Regulatory Network in Prostate Cancer with Bioinformatics Software. J Comput Biol.

[7] Ozgur E, Celik AI, Darendeliler E, Gezer U (2017). PCA3 silencing sensitizes prostate cancer cells to enzalutamide-mediated androgen receptor blockade. Anticancer Res.

[8] Gezer U, Tiryakioglu D, Bilgin E, Dalay N, Holdenrieder S (2015). Androgen stimulation of PCA3 and miR-141 and their release from prostate cancer cells. Cell J.

[9] Martinez-Pineiro L, Schalken JA, Cabri P, Maisonobe P, de la Taille A, Triptocare Study Group (2014). Evaluation of urinary prostate cancer antigen-3 (PCA3) and TMPRSS2-ERG score changes when starting androgen-deprivation therapy with triptorelin 6-month formulation in patients with locally advanced and metastatic prostate cancer. BJU Int.

[10] Ferreira LB, Palumbo A, de Mello KD, Sternberg C, Caetano MS, de Oliveira FL, Neves AF, Nasciutti LE, Goulart LR, Gimba ER (2012). PCA3 noncoding RNA is involved in the control of prostate-cancer cell survival and modulates androgen receptor signaling. BMC Cancer.

[11] Parolia A, Crea F, Xue H, Wang Y, Mo F, Ramnarine VR, Liu HH, Lin D, Saidy NR, Clermont PL, Cheng H, Collins C, Wang Y, Helgason CD (2015). The long non-coding RNA PCGEM1 is regulated by androgen receptor activity *in vivo*. Mol Cancer.

[12] Xue Y, Wang M, Kang M, Wang Q, Wu B, Chu H, Zhong D, Qin C, Yin C, Zhang Z, Wu D (2013). Association between lncrna PCGEM1 polymorphisms and prostate cancer risk. Prostate Cancer Prostatic Dis.

[13] Ifere GO, Ananaba GA (2009). Prostate cancer gene expression marker 1 (PCGEM1): a patented prostate- specific non-coding gene and regulator of prostate cancer progression. Recent Pat DNA Gene Seq.

[14] Coppola V, De Maria R, Bonci D (2010). MicroRNAs and prostate cancer. Endocr Relat Cancer.

[15] Shi XB, Tepper CG, White RW (2008). MicroRNAs and prostate cancer. J Cell Mol Med.

[16] Guo J, Huang X, Wang H, Yang H (2015). Celastrol Induces Autophagy by Targeting AR/miR-101 in Prostate Cancer Cells. PLoS One.

[17] Huang S, Guo W, Tang Y, Ren D, Zou X, Peng X (2012). miR-143 and miR-145 inhibit stem cell characteristics of PC-3 prostate cancer cells. Oncol Rep.

[18] Kristensen H, Haldrup C, Strand S, Mundbjerg K, Mortensen MM, Thorsen K, Ostenfeld MS, Wild PJ, Arsov C, Goering W, Visakorpi T, Egevad L, Lindberg J (2014). Hypermethylation of the GABRE∼miR-452∼miR-224 promoter in prostate cancer predicts biochemical recurrence after radical prostatectomy. Clin Cancer Res.

[19] Banyard J, Chung I, Wilson AM, Vetter G, Le Bechec A, Bielenberg DR, Zetter BR (2013). Regulation of epithelial plasticity by miR-424 and miR-200 in a new prostate cancer metastasis model. Sci Rep.

[20] Lynch SM, O’Neill KM, McKenna MM, Walsh CP, McKenna DJ (2016). Regulation of miR-200c and miR-141 by Methylation in Prostate Cancer. Prostate.

[21] Shi XB, Xue L, Ma AH, Tepper CG, Kung HJ, White RW (2011). miR-125b promotes growth of prostate cancer xenograft tumor through targeting pro-apoptotic genes. Prostate.

[22] Wang N, Li Q, Feng NH, Cheng G, Guan ZL, Wang Y, Qin C, Yin CJ, Hua LX (2013). miR-205 is frequently downregulated in prostate cancer and acts as a tumor suppressor by inhibiting tumor growth. Asian J Androl.

[23] Gandellini P, Giannoni E, Casamichele A, Taddei ML, Callari M, Piovan C, Valdagni R, Pierotti MA, Zaffaroni N, Chiarugi P (2014). miR-205 hinders the malignant interplay between prostate cancer cells and associated fibroblasts. Antioxid Redox Signal.

[24] Jalava SE, Urbanucci A, Latonen L, Waltering KK, Sahu B, Janne OA, Seppala J, Lahdesmaki H, Tammela TL, Visakorpi T (2012). Androgen-regulated miR-32 targets BTG2 and is overexpressed in castration-resistant prostate cancer. Oncogene.

[25] Xiang J, Bian C, Wang H, Huang S, Wu D (2015). MiR-203 down-regulates Rap1A and suppresses cell proliferation, adhesion and invasion in prostate cancer. J Exp Clin Cancer Res.

[26] Siu MK, Abou-Kheir W, Yin JJ, Chang YS, Barrett B, Suau F, Casey O, Chen WY, Fang L, Hynes P, Hsieh YY, Liu YN, Huang J, Kelly K (2014). Loss of EGFR signaling regulated miR-203 promotes prostate cancer bone metastasis and tyrosine kinase inhibitors resistance. Oncotarget.

[27] Hailer A, Grunewald TG, Orth M, Reiss C, Kneitz B, Spahn M, Butt E (2014). Loss of tumor suppressor mir-203 mediates overexpression of LIM and SH3 Protein 1 (LASP1) in high-risk prostate cancer thereby increasing cell proliferation and migration. Oncotarget.

[28] Viticchie G, Lena AM, Latina A, Formosa A, Gregersen LH, Lund AH, Bernardini S, Mauriello A, Miano R, Spagnoli LG, Knight RA, Candi E, Melino G (2011). MiR-203 controls proliferation, migration and invasive potential of prostate cancer cell lines. Cell Cycle.

[29] Saini S, Majid S, Yamamura S, Tabatabai L, Suh SO, Shahryari V, Chen Y, Deng G, Tanaka Y, Dahiya R (2011). Regulatory Role of mir-203 in Prostate Cancer Progression and Metastasis. Clin Cancer Res.

[30] Liu J, Uygur B, Zhang Z, Shao L, Romero D, Vary C, Ding Q, Wu WS (2010). Slug inhibits proliferation of human prostate cancer cells via downregulation of cyclin D1 expression. Prostate.

[31] Esposito S, Russo MV, Airoldi I, Tupone MG, Sorrentino C, Barbarito G, Di Meo S, Di Carlo E (2015). SNAI2/Slug gene is silenced in prostate cancer and regulates neuroendocrine differentiation, metastasis-suppressor and pluripotency gene expression. Oncotarget.

[32] Emadi Baygi M, Soheili ZS, Essmann F, Deezagi A, Engers R, Goering W, Schulz WA (2010). Slug/SNAI2 regulates cell proliferation and invasiveness of metastatic prostate cancer cell lines. Tumour Biol.

[33] Little GH, Baniwal SK, Adisetiyo H, Groshen S, Chimge NO, Kim SY, Khalid O, Hawes D, Jones JO, Pinski J, Schones DE, Frenkel B (2014). Differential effects of RUNX2 on the androgen receptor in prostate cancer: synergistic stimulation of a gene set exemplified by SNAI2 and subsequent invasiveness. Cancer Res.

[34] Liao H, Bai Y, Qiu S, Zheng L, Huang L, Liu T, Wang X, Liu Y, Xu N, Yan X, Guo H (2015). MiR-203 downregulation is responsible for chemoresistance in human glioblastoma by promoting epithelial-mesenchymal transition via SNAI2. Oncotarget.

[35] Atala A (2014). Re: miR-182 and miR-203 induce mesenchymal to epithelial transition and self-sufficiency of growth signals via repressing SNAI2 in prostate cells. J Urol.

[36] Ding X, Park SI, McCauley LK, Wang CY (2013). Signaling between transforming growth factor beta (TGF-beta) and transcription factor SNAI2 represses expression of microRNA miR-203 to promote epithelial-mesenchymal transition and tumor metastasis. J Biol Chem.

[37] Qu Y, Li WC, Hellem MR, Rostad K, Popa M, McCormack E, Oyan AM, Kalland KH, Ke XS (2013). MiR-182 and miR-203 induce mesenchymal to epithelial transition and self-sufficiency of growth signals via repressing SNAI2 in prostate cells. Int J Cancer.

[38] Zhang Z, Zhang B, Li W, Fu L, Fu L, Zhu Z, Dong JT (2011). Epigenetic Silencing of miR-203 Upregulates SNAI2 and Contributes to the Invasiveness of Malignant Breast Cancer Cells. Genes Cancer.

[39] Zhu M, Chen Q, Liu X, Sun Q, Zhao X, Deng R, Wang Y, Huang J, Xu M, Yan J, Yu J (2014). lncRNA H19/miR-675 axis represses prostate cancer metastasis by targeting TGFBI. FEBS J.

[40] Kang Y, Song J, Kim D, Ahn C, Park S, Chun CH, Jin EJ (2016). PCGEM1 stimulates proliferation of osteoarthritic synoviocytes by acting as a sponge for miR-770. J Orthop Res.

[41] Tiryakioglu D, Bilgin E, Holdenrieder S, Dalay N, Gezer U (2013). miR-141 and miR-375 induction and release are different from PSA mRNA and PCA3 upon androgen stimulation of LNCaP cells. Biomed Rep.

[42] Zhang Z, Zhou N, Huang J, Ho TT, Zhu Z, Qiu Z, Zhou X, Bai C, Wu F, Xu M, Mo YY (2016). Regulation of androgen receptor splice variant AR3 by PCGEM1. Oncotarget.

[43] Boll K, Reiche K, Kasack K, Morbt N, Kretzschmar AK, Tomm JM, Verhaegh G, Schalken J, von Bergen M, Horn F, Hackermuller J (2013). MiR-130a, miR-203 and miR-205 jointly repress key oncogenic pathways and are downregulated in prostate carcinoma. Oncogene.

[44] Sun T, Yang M, Chen S, Balk S, Pomerantz M, Hsieh CL, Brown M, Lee GM, Kantoff PW (2012). The altered expression of MiR-221/-222 and MiR-23b/-27b is associated with the development of human castration resistant prostate cancer. Prostate.

[45] Wu K, Gore C, Yang L, Fazli L, Gleave M, Pong RC, Xiao G, Zhang L, Yun EJ, Tseng SF, Kapur P, He D, Hsieh JT (2012). Slug, a unique androgen-regulated transcription factor, coordinates androgen receptor to facilitate castration resistance in prostate cancer. Mol Endocrinol.

[46] Jing Y, Cui D, Guo W, Jiang J, Jiang B, Lu Y, Zhao W, Wang X, Jiang Q, Han B, Xia S (2014). Activated androgen receptor promotes bladder cancer metastasis via Slug mediated epithelial-mesenchymal transition. Cancer Lett.

[47] Eisermann K, Fraizer G (2017). The Androgen Receptor and VEGF: Mechanisms of Androgen-Regulated Angiogenesis in Prostate Cancer. Cancers (Basel).

[48] Russo MA, Ravenna L, Pellegrini L, Petrangeli E, Salvatori L, Magrone T, Fini M, Tafani M (2016). Hypoxia and Inflammation in Prostate Cancer Progression. Cross-talk with Androgen and Estrogen Receptors and Cancer Stem Cells. Endocr Metab Immune Disord Drug Targets.

[49] Colombel M, Filleur S, Fournier P, Merle C, Guglielmi J, Courtin A, Degeorges A, Serre CM, Bouvier R, Clezardin P, Cabon F (2005). Androgens repress the expression of the angiogenesis inhibitor thrombospondin-1 in normal and neoplastic prostate. Cancer Res.

[50] Leisegang MS, Fork C, Josipovic I, Richter FM, Preussner J, Hu J, Miller MJ, Epah J, Hofmann P, Gunther S, Moll F, Valasarajan C, Heidler J (2017). Long Noncoding RNA MANTIS Facilitates Endothelial Angiogenic Function. Circulation.

