# Supplementary material for: The PCAT3/PCAT9-miR-203-SNAI2 axis functions as a key mediator for prostate tumor growth and progression
Source: Oncotarget. 2018 Jan 12;9(15):12212–25. doi: 10.18632/oncotarget.24198 (PMC5844740; doi:10.18632/oncotarget.24198)
Supplement: Supplementary file 1 [file oncotarget-09-12212-s001.pdf]

The PCAT3/PCAT9-miR-203-SNAI2 axis functions as a key mediator for prostate tumor growth and progression

SUPPLEMENTARY MATERIALS

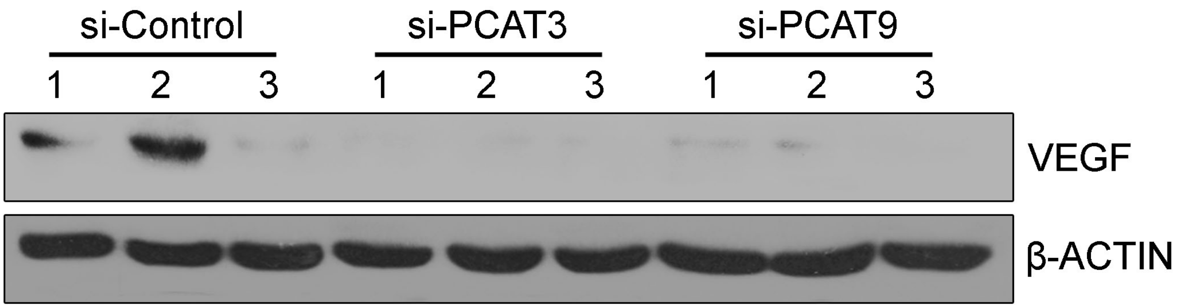

Supplementary Figure 1: VEGF expression is decreased in xenograft tissues with PCAT3 or PCAT9 siRNA transfection. Three tumors were subjected to western blot analysis for each group.
